# Association between C-Reactive Protein to Albumin Ratio and Multi-Vessel Coronary Artery Disease in Patients with Stable Coronary Artery Disease

**DOI:** 10.3390/jpm14040378

**Published:** 2024-03-31

**Authors:** Suleyman Akkaya, Umit Cakmak

**Affiliations:** 1 Department of Cardiology, Health Sciences University, Gazi Yasargil Research and Training Hospital, 21070 Diyarbakir, Turkey; 2 Department of Nephrology, Health Sciences University, Gazi Yasargil Research and Training Hospital, 21070 Diyarbakir, Turkey; umit.cakmak@memorial.com.tr

**Keywords:** stable angina pectoris, coronary artery disease severity, albumin, C-reactive protein, C-reactive protein/albumin ratio

## Abstract

Multivessel coronary artery disease (MV-CAD) remains a prevalent and serious health concern despite advances in treatment. Early identification and risk stratification are crucial for optimizing treatment. The CRP-to-albumin ratio (CAR) has emerged as a promising biomarker in various inflammatory diseases. This study investigated the potential of CAR as a marker for MV-CAD. We retrospectively analyzed 1360 patients with suspected CAD. Patients were divided into three groups based on CAR tertiles. Logistic regression analyses were carried out to estimate the association between MHR and MV-CAD. Elevated CAR levels were significantly associated with an increased prevalence of CAD (*p* < 0.001), severe CAD (*p* < 0.001), and MV-CAD (*p* < 0.001). Patients with the highest CAR tertile had five times higher odds of MV-CAD compared to the lowest tertile (*p* < 0.001). CAR demonstrated moderate accuracy in predicting MV-CAD (AUC: 0.644, 95% CI: 0.615–0.674, *p* < 0.001). CAR holds promise as a tool for the early identification and risk stratification of multivessel CAD. Further research is warranted to validate its clinical utility and explore its potential to guide treatment decisions and improve outcomes in patients with this high-risk condition.

## 1. Introduction

Despite improved survival rates for coronary artery disease (CAD) in recent years, CAD overall prevalence remains a significant concern [1]. With the growing number of older people, along with rising rates of diabetes and obesity, multivessel CAD (MV-CAD) accounts for 30–40% of patients with CAD [2]. Compared to those with single-vessel CAD, MV-CAD patients tend to have more severe complications, including left ventricular dysfunction, comorbidities, and, unfortunately, a higher risk of death [3,4]. The optimal revascularization strategy for MV-CAD patients depends on a detailed assessment of several factors, including the extent and severity of coronary artery blockages, the presence and burden of comorbidities such as diabetes or heart failure, and the anticipated risk-benefit profiles of both coronary artery bypass grafting (CABG) and percutaneous coronary intervention (PCI) [5,6]. Due to the significant burden of MV-CAD, marked by its prevalence and dismal prognosis, identifying biological markers for early detection is crucial to optimizing surgical methods and improving treatment outcomes for this high-burden condition.

Chronic inflammation is a critical pathophysiological component of atherosclerosis, marked by endothelial cell injury, hemodynamic disturbances, and dysregulated lipid metabolism in early-stage atherosclerosis [7,8]. This pathology is exacerbated by flow-mediated inflammatory alterations in the endothelium, a critical layer in vascular biology [9]. Consequently, the role of inflammatory biomarkers is being intensively studied for their ability to determine the prognosis and severity of patients with CAD [10]. A key biomarker in this field is C-reactive protein (CRP), a prototypical marker of systemic inflammation, which not only plays a contributory role in the pathophysiology of CAD but also holds significant clinical interest as a marker for disease assessment and prognosis [11,12]. Furthermore, observational studies have demonstrated an association between hypoalbuminemia and the development of CAD [13]. In the population of patients with CAD, hypoalbuminemia has been identified as a predictor of future myocardial infarction (MI) events [14]. Studies have shown that the CRP-to-albumin ratio (CAR), a relatively new measure of inflammation, often outperforms both CRP and albumin alone in predicting disease severity, mortality, and other clinical outcomes in various inflammatory diseases such as sepsis, cancer, acute pancreatitis, ulcerative colitis, and hepatitis B [15,16]. Despite recent evidence highlighting the potential of CAR as a marker for CAD and prognostic indicator in percutaneous coronary intervention (PCI) (refs. [12,17]), no prior study has investigated the association between CAR and the complexity of vessel lesions in CAD patients, specifically focusing on MV-CAD. Therefore, this study comprehensively investigated the association between CAR and MV-CAD.

## 2. Methods

### 2.1. Study Population

A retrospective study analyzed electronic medical records from the Gazi Yasargil Research and Training Hospital cardiology department, focusing on 1360 patients suspected of CAD. Inclusion criteria encompassed individuals presenting with: (1) typical angina-like chest pain or tightness, (2) abnormalities in myocardial enzymes or troponin levels suggestive of myocardial injury, or (3) electrocardiogram (ECG) findings indicative of potential ischemia. Patients with previous PCI or CABG procedures, missing or incomplete C-reactive protein (CRP) and albumin levels, active infections, or connective tissue disorders were excluded. The study protocol was approved by the Ethics Committee of Health Sciences University Gazi Yasargil Hospital in accordance with the declaration of Helsinki, and written informed consent was obtained from all participants.

### 2.2. Coronary Angiography

The study employed coronary angiography via the radial artery to assess the degree of stenosis in four major coronary arteries: the left main coronary artery (LMCA), left anterior descending (LAD), right coronary artery (RCA), and left circumflex artery (LCx). CAD was defined as stenosis exceeding 50% in any of these arteries. Severe CAD was defined as stenosis ≥50% in the LM or ≥70% in any other major artery. Multi-vessel CAD was diagnosed if stenosis affected the epicardial segment of more than one major artery. Conversely, single-vessel CAD involved stenosis in only one major artery [5].

### 2.3. Laboratory Measurements

Pre-angiography blood samples were analyzed at our institution’s laboratory for a complete blood count, lipid profile (LDL-C, HDL-C, triglycerides), albumin, and CRP. The serum albumin level was analyzed using automatic photometry commercial kits using Abbott C8000i (Abbott Park, Illinois, USA). Serum CRP levels were measured with a nephelometric method (UniCel DxC 800 System; Beckman Coulter Inc, Brea, California, USA). The C-Reactive Protein to Albumin Ratio (CAR) was then calculated by dividing CRP by albumin.

### 2.4. Statistical Analysis

Descriptive statistics were represented as the median, interquartile range (IQR; 25th–75th percentile) and standard errors for continuous variables and frequency and percentages for categorical variables. The results of the three groups were compared using variance analysis or the χ^2^ test for categorical variables and the Kruskal–Wallis test for continuous variables. Multivariate analyses were conducted by logistic regression analyses via backward variable selection, and odds ratios were calculated together with 95% confidence intervals (CI). Clinical and laboratory parameters with *p*-values < 0.10 were included in the multivariate model. All statistical analyses were performed in SPSS, version 25.0 (IBM Inc., Armonk, NY, USA), and a *p* value of less than 0.05 was considered statistically significant.

## 3. Results

This study recruited 1360 patients who met the inclusion and exclusion criteria. The patients were divided into three groups based on CAR, with 451 patients in the low CAR group (<0.57), 453 patients in the medium CAR group (0.57–1.18), and 456 patients in the high CAR group (>1.18) (Table 1). Patients with high CAR showed an older age and a higher incidence of diabetes and had higher levels of LDL, white blood cell count, neutrophil count, CRP level in contrast as well as lower HDL cholesterol and albumin levels. Some baseline characteristics were similar across all CAR tertiles, including gender, hypertension, triglycerides, hemoglobin and lymphocyte count.

**Figure 1:**
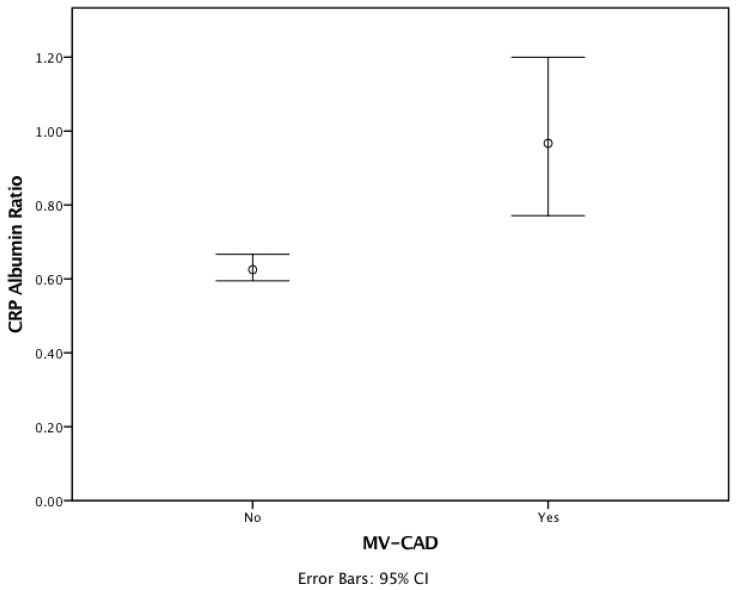
Distribution of CAR levels according to multi-vessel involvement.

Out of 1360 patients, 732 had CAD, 382 had severe CAD, and 349 had multivessel CAD (MV-CAD). The prevalence of all three types of CAD significantly increased with higher CAR levels (*p* < 0.001 for each). Severe-CAD patients were subgrouped by CAR tertiles, with 81 patients in the low CAR group (<0.57), 138 patients in the medium CAR group (0.57–1.18), and 163 patients in the high CAR group (>1.18) (Table 2). With the exception of age, lymphocyte count, neutrophil count, albumin, LDL-C and CRP levels, no significant differences were observed between tertiles. Among the 382 patients with severe CAD, 81 had single-vessel CAD (SV-CAD) and 320 had MV-CAD. Notably, MV-CAD was significantly more prevalent in the highest CAR tertile (*p* < 0.001). Additionally, mean serum CAR levels were significantly higher in patients with MV-CAD compared to non-MV-CAD (*p* < 0.001) (Figure 1).

**Figure 2:**
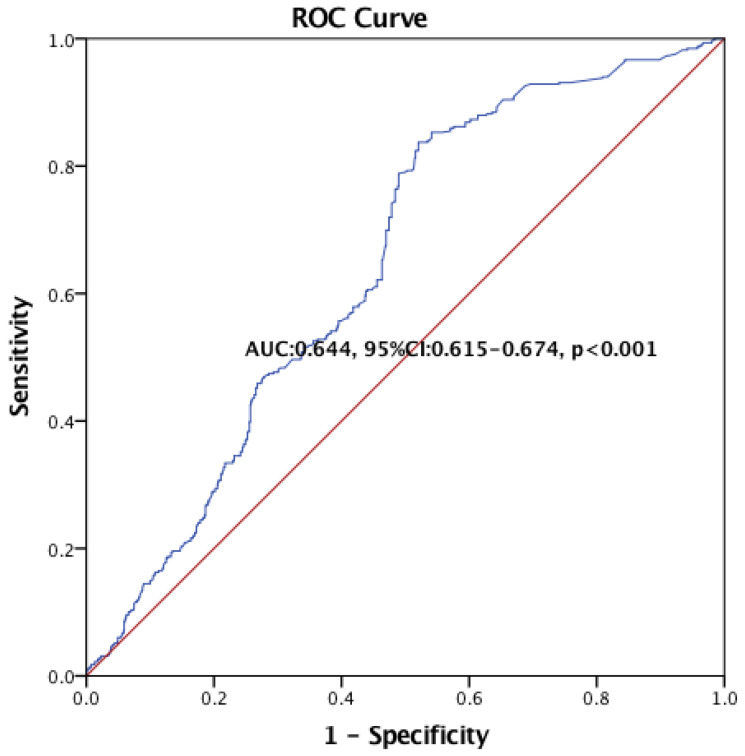
ROC curve analysis for CAR reflected MV-CAD in suspected CAD patients.

Multivariate regression analysis revealed a statistically significant association between CAR levels and MV-CAD in both crude and adjusted models for suspected CAD patients (*p* < 0.009 and *p* < 0.007, respectively). Furthermore, the number of patients with severe CAD and MV-CAD progressively increased across CAR tertiles. Compared with the control group, the OR (95% CI) values of the mid-tertile and the high tertile were 2.00 (1.46–2.73) and 2.54 (1.86, 3.45), respectively (*p* for trend < 0.001). This association remained statistically significant even after adjusting for potential confounding factors. The positive relationship between CAR and MV-CAD was confirmed in suspected CAD patients (both crude and adjusted *p* for trend < 0.001) (Table 3). The CAR had moderate success for the prediction of MV-CAD (AUC: 0.644, 95% CI: 0.615–0.674, *p* < 0.001) (Figure 2).

## 4. Discussion

The present study indicated that higher CAR levels were significantly associated with an increased prevalence of severe CAD and MV-CAD after adjusting for potential confounding factors. Patients with MV-CAD had significantly higher CAR levels compared to those with single-vessel CAD, suggesting a potential role of CAR in differentiating the severity of CAD. Patients in the highest CAR tertile had five times higher odds of MV-CAD compared to the lowest group, highlighting the potential of CAR as a prognostic marker for this high-risk population.

Recognizing the critical role of inflammation in atherosclerosis, clinicians frequently utilize markers reflecting inflammation, such as CRP and albumin [18]. Serum CRP, an acute-phase protein produced by the liver, holds particular significance due to its ability to both reflect inflammation and improve risk prediction for patients with CAD [19]. Beyond its role as an inflammation marker, elevated CRP may directly contribute to CAD severity through various mechanisms [20]. It can hinder endothelial repair, impair fibrinolysis, enhance collagen degradation in immune cells, and potentially enhance LDL uptake by macrophages, turning them into foam cells [21]. Hypoalbuminemia may portend a heightened risk of adverse outcomes, both morbidity and mortality, in a myriad of cardiovascular diseases [22]. This association is corroborated by evidence from numerous studies, including findings by Kurtul et al., who demonstrated hypoalbuminemia as an independent predictor of both elevated ST-segment elevation and in-hospital mortality in patients with ACS [23]. Beyond reflecting the chronic nature of the disease, hypoalbuminemia serves as a surrogate marker for inflammatory status. Furthermore, a compelling body of research suggests a significant association between hypoalbuminemia and several factors known to exacerbate CAD severity, including elevated blood viscosity, impaired endothelial function, heightened platelet activation and aggregation, and increased synthesis of platelet-derived growth factor (PDGF), a key mediator of coronary artery stenosis [24,25]. These intertwined pathophysiological mechanisms may provide the underpinnings for the observed link between serum albumin levels and CAD severity.

CAR encompasses measurements of both CRP and albumin, thereby being a more comprehensive reflection of the pro-inflammatory state as well as the nutritional status of a patient. Previous research has established that in patients with cancer, CAR not only serves as a prognostic tool but also rises in tandem with disease progression. Consequently, CAR provides a more reliable biomarker for assessing the severity and prognosis of various diseases compared to evaluating CRP and albumin levels independently. Furthermore, an association has been observed between CAR and all-cause mortality in individuals diagnosed with malignant tumors [26]. Recent studies that evaluated CAR in patients with CAD showed encouraging results. In a study that included patients with STEMI, researchers found that the white blood cell count, neutrophil-to-lymphocyte ratio, and CAR were associated with a no-reflow phenomenon. They also found that after performing ROC curve analysis, CAR had higher AUC values compared to CRP and albumin levels separately [12]. A recent meta-analysis demonstrated a significant association between a high CAR value and poor outcomes in patients with acute and chronic HF [27]. This included an increased risk of all-cause mortality. While the CAR value was significantly higher in patients with a worse New York Heart Association (NYHA) functional class, it did not show a correlation with left ventricular ejection fraction (LVEF). Additionally, high CAR levels were associated with a greater likelihood of complications, organ failure, and the need for interventions. Birdal et al. revealed that a higher CAR level at hospitalization was found to be associated with long-term mortality in ACS patients [28]. CAR demonstrates a prognostic value not only for predicting future mortality but also for identifying patients at risk of developing advanced HF, albeit with moderate diagnostic accuracy. Notably, HF is known to worsen multiorgan function, and this study observed a higher degree of multiorgan dysfunction in the high-CAR group compared to the low-CAR group [29]. Calik et al. demonstrated that in patients undergoing iliac artery stent implantation, a higher CAR value predicted an increased risk of in-stent restenosis [30]. Also, Cinar et al. found that CAR is an independent predictor of all-cause mortality in patients with ST elevation myocardial infarction [31]. In light of these studies, this study specifically demonstrated a robust association between CAR and MV-CAD. Among suspected CAD patients, an association was observed between CAR and CAD, Severe-CAD, and MV-CAD. CAD risk was increased in patients with higher CAR, which was also observed in Severe-CAD and MV-CAD patients.

While this study demonstrates a promising link between CAR and MV-CAD, it is crucial to acknowledge limitations inherent in relying solely on a single spot value for CAR. CAR reflects a patient’s inflammatory state at a specific point in time [32]. However, inflammatory markers can fluctuate due to various factors such as recent infections or acute illness [33]. Additionally, chronic inflammatory conditions beyond atherosclerosis can also elevate CAR [34]. Future research should explore the impact of serial CAR measurements over time to assess its effectiveness in capturing a patient’s overall inflammatory burden and its association with MV-CAD progression. This could potentially lead to a more nuanced understanding of how CAR can be used for risk stratification and potentially guide treatment decisions.

Our study had some limitations; first of all, the retrospective design may inherently limit the causal inferences that can be drawn from the findings. Secondly, because people who had already received bypass surgery or stents were not included, the study may not fully represent the whole group of people with MV-CAD. Thirdly, patient data were collected from a single site, limiting the applicability of the results to other communities, and additional research is required to verify these findings. Moreover, the use of a single spot value for CAR limits the study’s ability to consider potential fluctuations in this marker, which might influence the accuracy of the associations observed. Lastly, unfortunately, data on body mass index (BMI) and smoking status were not available in our dataset. This limits our ability to fully assess the influence of these established CAD risk factors on the observed association between CAR and MV-CAD. Nevertheless, there are some strengths in the clinical use of CAR. CAR uses routinely measured laboratory values, CRP and albumin, making it a relatively inexpensive and accessible tool. Compared to CRP or albumin alone, CAR provides a more comprehensive assessment of both inflammatory status and nutritional status. This composite measure may offer a more accurate reflection of the patient’s risk profile, particularly for MV-CAD, as demonstrated in this study.

## 5. Conclusions

Our study found a strong link between elevated CAR levels and the presence and extent of coronary artery disease, particularly multivessel CAD. This suggests that CAR could be a valuable biomarker for identifying not only the presence of CAD but also its severity, potentially aiding in risk stratification and guiding treatment decisions. Furthermore, CAR demonstrated moderate accuracy in predicting MV-CAD, paving the way for its potential use in clinical practice.

## Figures and Tables

**Table 1:** Baseline characteristics and coronary angiography results of patients with suspected CAD (*n* = 1360).

Variables	The Level of CAR						
	<0.57 (*n* = 451)	0.57–1.18 (*n* = 453)	>1.18 (*n* = 456)	*p* Value Low vs. Medium	*p* Value Low vs. High	*p* Value Medium vs. High	*p* Value Overall
Age (years)	57 (49–65)	59 (51–68)	59 (50–69)	0.003	0.011	0.699	0.015
Gender, male *n*, (%)	240 (53.2)	233 (51.4)	239 (52.4)	0.592	0.412	0.768	0.866
Diabetes mellitus, *n*, (%)	78 (17.3)	94 (20.8)	109 (23.9)	0.186	0.089	0.254	0.049
Hypertension, *n*, (%)	152 (33.7)	170 (37.5)	161 (35.3)	0.230	0.389	0.487	0.483
**Laboratory findings**							
Triglycerides (mg/dL), median (IQR)	138 (100–211)	151 (100–217)	145 (102–223)	0.142	0.148	0.868	0.800
HDL-C (mg/dL), median (IQR)	44 (36–52)	43 (36–50)	42 (36–50)	0.096	0.028	0.519	0.018
LDL-C (mg/dL), median (IQR)	106 (87–133)	106 (84–132)	110 (88–133)	0.577	0.335	0.132	0.209
Hemoglobin (g/dL), median (IQR)	13.70 (11.70–15.10)	13.60 (11.90–15.10)	13.60 (11.90–14.90)	0.064	0.410	0.284	0.795
White cell count (10^3^/mL), median (IQR)	8.36 (6.94–10.39)	8.43 (7.09–10.09)	8.83 (7.40–10.47)	0.407	0.589	0.177	0.085
Neutrophil count (10^3^/mL), median (IQR)	5.12 (4.17–6.87)	5.65 (4.27–7.11)	5.65 (4.46–7.53)	0.171	0.229	0.827	0.020
Lymphocyte count (10^3^/mL), median (IQR)	2.16 (1.49–2.79)	2.14 (1.55–2.84)	2.20 (1.59–2.82)	0.337	0.199	0.810	0.819
Platelet count (10^3^/mL), median (IQR)	257 (211–299)	258 (217–305)	260 (218–312)	0.488	0.171	0.487	0.879
Albumin (g/dL), median (IQR)	4.20 (4.0–4.50)	4.10 (3.70–4.40)	4.10 (3.70–4.40)	<0.001	<0.001	0.678	<0.001
CRP, (mg/L), median (IQR)	2 (2.0–2.10)	2.80 (2.40–3.50)	10 (7.0–25.50)	<0.001	<0.001	<0.001	<0.001
**Coronary angiography results**							
CAD, *n* (%)	209 (46.3)	243 (53.6)	280 (61.4)	0.028	<0.001	0.018	<0.001
Severe-CAD, *n* (%)	81 (18.0)	138 (30.5)	163 (35.7)	<0.001	<0.001	0.091	<0.001
MV-CAD, *n* (%)	63 (14.0)	180 (39.7)	206 (45.2)	<0.001	<0.001	0.097	<0.001

**Abbreviations:** LVEF: Left ventricular ejection fractions, LDL-C: Low-density lipoprotein cholesterol, HDL-C: High-density lipoprotein cholesterol, CAD: Coronary artery disease, Severe-CAD: severe coronary artery disease, SV-CAD: Single-vessel coronary artery disease, MV-CAD: Multi-vessel coronary artery disease.

**Table 2:** Baseline characteristics of patients with Severe-CAD.

Variables	The Level of CAR			*p* Value
	<0.57 (*n* = 81)	0.57–1.18 (*n* = 138)	>1.18 (*n* = 163)	
Age (years)	59 (52–66)	66 (58–72)	63 (55–71)	0.002
Gender, male *n*, (%)	54 (66.7)	90 (65.2)	101 (41.2)	0.730
Diabetes mellitus, *n*, (%)	21 (25.9)	39 (28.3)	50 (30.7)	0.731
Hypertension, *n*, (%)	33 (40.7)	74 (53.6)	76 (46.6)	0.167
**Laboratory findings**				
Triglycerides (mg/dL), median (IQR)	151 (107–216)	150 (101–221)	154 (106–218)	0.977
HDL-C (mg/dL), median (IQR)	42 (35–54)	41 (36–47)	42 (35–50)	0.816
LDL-C (mg/dL), median (IQR)	103 (88–138)	96 (79–120)	108 (89–130)	0.011
Hemoglobin (g/dL), median (IQR)	13.60 (−11.90–14.91)	14.20 (12.60–15.20)	13.80 (12.30–15.20)	0.591
White blood cell count (10^3^/mL), median (IQR)	8.40 (7.50–9.62)	8.54 (7.04–9.96)	9.09 (7.63–10.82)	0.140
Neutrophil count (10^3^/mL), median (IQR)	4.87 (4.18–6.19)	5.69 (4.41–6.78)	6.21 (5.21–7.61)	<0.001
Lymphocyte count (10^3^/mL), median (IQR)	1.82 (1.36–2.51)	2.04 (1.54–2.84)	2.37 (1.72–2.92)	0.005
Platelet count (10^3^/mL), median (IQR)	238 (198–293)	239 (203–296)	256 (212–315)	0.224
Albumin (g/dL), median (IQR)	4.20 (4.10–4.60)	4.10 (3.80–4.30)	4.00 (3.50–4.30)	0.001
CRP, (mg/L), median (IQR)	2.0 (2.0–2.0)	2.8 (2.70–3.0)	4.0 (3.5–4.30)	<0.001
**Coronary angiography results**				
SV-CAD, *n* (%)	35 (43.2)	17 (12.3)	16 (9.8)	<0.001
MV-CAD, *n* (%)	46 (14.6)	121 (38.5)	147 (46.8)	<0.001

**Table 3:** Multivariable-adjusted association of CAR and coronary angiography results in patients who underwent coronary angiography.

Variables		Crude			Adjusted		
		OR	95%CI	*p* Value	OR	95%CI	*p* Value
Association between CAR and Severe-CAD							
CAR							
	Continuous	1.015	0.997–1.032	0.096	1.015	0.997–1.034	0.108
	Tertiles						
	<0.57	1			1		
	0.57–1.18	2.001	1.464–2.736	<0.001	1.874	1.346–2.608	<0.001
	>1.18	2.541	1.869–3.456	<0.001	2.494	1.803–3.452	<0.001
Association between CAR and MV-CAD							
CAR							
	Continuous	1.025	1.006–1.044	0.009	1.029	1.008–1.051	0.007
	Tertiles						
	<0.57	1			1		
	0.57–1.18	4.061	2.931–5.626	<0.001	4.355	3.061–6.197	<0.001
	>1.18	5.075	3.671–7.016	<0.001	5.620	3.958–7.982	<0.001

Crude, no adjustment; Adjusted: age, gender, diabetes, hypertension, neutrophil count, lymphocyte count, triglyceride and LDL-C were adjusted.

## Data Availability

The datasets used and/or analyzed during the current study are available from the corresponding author on reasonable request.
